# Ergocalciferol treatment does Not improve erythropoietin utilization and hospitalization rate in hemodialysis patients

**DOI:** 10.1186/s12882-016-0359-7

**Published:** 2016-10-07

**Authors:** Gaurav Agarwal, Padam Hirachan, Jonathan Gelfond, Paolo Fanti, Claudia Hura, Shweta Bansal

**Affiliations:** 1 Department of Medicine, Division of Nephrology, University of Alabama at Birmingham School of Medicine, Birmingham, AL USA; 2 Department of Medicine, Division of Nephrology, University of Texas Health Sciences Center at San Antonio, 7703 Floyd Curl Drive, MSC 7882, San Antonio, TX 78229 USA; 3 Department of Epidemiology and Biostatistics, University of Texas Health Sciences Center at San Antonio, San Antonio, TX USA; 4 Renal Section, South Texas Veterans HealthCare System, San Antonio, TX USA

**Keywords:** Hemodialysis, Vitamin D deficiency, Ergocalciferol, EPO requirement, Hospitalization

## Abstract

**Background:**

Vitamin D (25-hydroxyvitamin D; 25[OH]D) deficiency (VDD) is highly prevalent in chronic kidney disease. The aim of this study was to evaluate the effect of oral ergocalciferol supplementation on requirement of erythropoietin (EPO) and active vitamin D analogues, and hospitalization rate in maintenance hemodialysis (HD) patients.

**Methods:**

This retrospective cohort study included 186 patients who were on HD for 3 months and had 25(OH)D levels < 30 ng/ml. Over 1-year period, 107 patients were supplemented with protocol-based ergocalciferol (D_2_ group) and 79 were not (control). Parameters of erythropoiesis and bone-mineral metabolism, and monthly doses of EPO and paricalcitol were assessed at 6- and 12- months of ergocalciferol supplementation. Total hospitalizations were recorded for the same year.

**Results:**

Baseline characteristics were similar across two arms except higher serum ferritin, transferrin saturation and prevalence of stroke in D2 and higher coronary artery disease in control group with overall mean ± SD 25(OH)D level of 16.8 ± 7 ng/ml. At 12 months, 25(OH)D levels increased significantly in D_2_ group compared to control (30.5 ± 11.7 vs. 14.2 ± 9.3 ng/ml; *p* < 0.001). The EPO dose remained same with no difference in hemoglobin values between the two groups during the intervention period. On multivariate regression which included above variables there was no effect of ergocalciferol treatment on EPO dose (*p* = ns). Hospitalization rate was similar in two arms; however, ergocalciferol treatment inversely associated with paricalcitol dose (β ± SE = −10.4 ± 2.8; *p* < 0.001).

**Conclusions:**

One-year of ergocalciferol supplementation was not associated with reduction in EPO requirement or hospitalization rate in HD patients with VDD. Further studies are warranted to determine definitive role of nutritional vitamin D in these patients.

## Background

Vitamin D deficiency (VDD) has prevalence as high as 92 % in patients with chronic kidney disease (CKD) and end-stage renal disease (ESRD) and it has been associated with decreased muscle strength, anemia, resistance to treatment with recombinant human erythropoietin (EPO) and increased mortality in these patients [1–6]. The Kidney Disease Improving Global Outcome (KDIGO) initiative recommends that nutritional vitamin D is used to correct VDD in addition to active forms of vitamin D in ESRD patients [7]. Active vitamin D, either as the native hormone 1,25-dihydroxy vitamin D (1,25(OH)_2_D) or as one of its synthetic analogs (paricalcitol, doxercalciferol, etc.), is conventionally used to correct low levels of 1,25(OH)_2_D that are caused by loss of renal 1-α-hydroxylase enzyme function. The KDIGO rationale for recommending nutritional vitamin D supplementation is based on the emerging notions that the 1-α-hydroxylase enzyme remains active in the extra-renal tissues of ESRD patients and that even low amounts of extra-renally produced 1,25(OH)_2_D may be important for local tissue function [8]. Basic research has shown that by binding to ubiquitous vitamin D receptor, locally produced 1,25(OH)_2_D exerts autocrine and paracrine effects including regulation of cellular proliferation and differentiation, modulation of immune system function, inhibition of renin synthesis and insulin production, etc.[9].

Anemia is a common complication of ESRD, primarily the consequence of reduced EPO production and of uremia- and resultant hyperparathoyroidism-related resistance to this hormone. It has been suggested that nutritional vitamin D may be effective in reversing EPO resistance via effects of extra-renally produced 1,25(OH)_2_D on inflammation and insulin resistance. Recent clinical studies have suggested that use of nutritional vitamin D may lower the dose requirement of EPO simulating agents (ESA) in ESRD patients on maintenance hemodialysis (HD) [2, 10–12]; none of these studies, however, had a control arm. We present here a one-year clinical study with primary aim to examine the effect of ergocalciferol supplementation on EPO dose requirements in maintenance HD patients with VDD. Secondary objectives were to monitor the effect of ergocalciferol supplementation on parameters of mineral metabolism and on the rate of hospitalization.

## Methods

### Study design

The study had a retrospective cohort design with 1-year follow-up in prevalent HD patients from three free-standing dialysis clinics that are located in the same urban area, managed by same administration and staffed by nephrologists of the University of Texas Health Sciences Center at San Antonio who follow the same policies and practices. The study protocol was approved by the local institutional review board and is in accordance with the ethical standards of the Declaration of Helsinki.

### Study population

Medical records of all 239 ESRD patients receiving HD at the three clinics between January 2011 and January 2012 were reviewed. Patients were included in the study if they were 18 years of age or older, initiated dialysis at least three months prior to January 2011, received HD treatment thrice weekly, had the serum 25-hydroxy vitamin D [25(OH)D] level checked with VDD, i.e., 25(OH)D level < 30 ng/ml. After exclusion of 40 subjects for < 3 months enrollment in the HD program and 13 subjects for 25(OH)D levels ≥ 30 ng/ml, the study population included a total of 186 patients.

#### Ergocalciferol (D_2_) vs control group

Following the KDIGO-2009 guidelines which suggest that in CKD 3-5D, 25(OH)D levels might be measured and VDD and insufficiency be corrected using treatment strategies recommended for the general population (2C level) [7], University nephrology practice started measuring the 25(OH)D levels in 2010 and ergocalciferol capsules were purchased in the bulk to be administered by the nursing staff to the patients with VDD during HD treatment to avoid the need for filling the prescription. Due to some administrative and technical difficulties, only one of the three dialysis clinics could start ergocalciferol administration in January 2011, and the other two units started in 2012. One-hundred-seven VDD patients from the first dialysis clinic received oral ergocalciferol with dosing and schedule as recommended by KDIGO (Table 1; D_2_ group); whereas, 79 VDD patients from the other two dialysis units served as control group, i.e., they were subjected to the same monitoring as the D_2_ group but without ergocalciferol treatment.

**Table 1:** Ergocalciferol Supplementation Protocol

25(OH)D	Ergocalciferol dose	Duration (months)	Comment
<5 ng/ml	50,000 IU PO/Week x 12 weeks, then monthly	6	Measure 25-OH D after 6 month and repeat course based on level
5–15 ng/ml	50,000 IU PO/Week x 4 weeks, then monthly	6	Same as above
16–30 ng/ml	50,000 IU PO/Month orally	6	Same as above

### Measurement of demographic, biochemical and clinical data

Information collected at the beginning of the study included demographics, history of diabetes, hypertension, cardiovascular disease, stroke, cancer and dialysis vintage. Laboratory data were collected at baseline, 6 and 12 months, and included hemoglobin, serum iron, total iron binding capacity, ferritin, calcium, phosphorus, albumin, and intact parathyroid hormone (iPTH). Information was collected regarding monthly administered dose of EPO (IU/month), active vitamin D analog paricalcitol (μg/month) and iron sucrose (mg/month) for the same three time periods. Adjustments of EPO and iron dose were performed by staff nephrologists with target hemoglobin 10–12 g/dL during early period of the study. However, target hemoglobin was changed to 10–11 g/dL during the middle of the study in accordance with centers of Medicare and Medicaid services (CMS) guidelines. Adjustments of paricalcitol dose were made to keep iPTH between 150 and 400 ng/ml. Serum 25(OH)D levels were analyzed with a chemiluminescence assay (Arup, Salt Lake City, Utah) which measures the combination of 25(OH)D_2_ and 25(OH)D_3_ levels. Other lab parameters were measured using standard techniques at the University Hospital Centralized Clinical Laboratory.

#### Follow-up cohort

In 2012, the other two units also started ergocalciferol supplementation following the same protocol. We collected same information including loboratory data, monthly administered EPO, paricalcitol and venofer doses in the whole study population for the next 2 years.

### Statistical analysis

Summary statistics for demographic, dosing, and laboratory variables were computed as follows: continuous variables were described using mean ± standard deviation (SD) or median with interquartile ranges (IQR) as appropriate, while categorical variables were described with percentages. Continuous variable were accessed using Mann Whitney *U* Test. The categorical variables were compared using Chi-square test. For continuous laboratory measures, we fit a linear mixed-effect model with time and treatment effects in order to identify time trends within the follow-up period and associations with treatment.

We examined the effects of multiple variables on EPO and paricalcitol doses simultaneously using multiple regression within a linear mixed-effect model. We considered demographics, comorbidities, and laboratory values as predictors alongside ergocalciferol treatment. Because the number of predictor variables was large, we included variables in the mixed-effect regression if these had univariate associations with the outcome (*p* < 0.1). To identify temporal associations with treatment, we tested the Time x Treatment interaction. This interaction assesses the effect of ergocalciferol supplementation on the monthly EPO or paricalcitol dose depending on time/duration of follow-up, i.e., at month 6 and 12. We performed logistic regression to determine effect of ergocalciferol treatment on the probability of hospitalization (hospitalization or not). We considered the demographics, baseline comorbidities and laboratory measures as potential predictors of hospitalizations, and included these in the logistic regression if there was evidence of a univariate association (*p* < 0.1) with the outcome. All calculations were done using R software (Version 3+, Vienna, Austria).

#### Outcomes

The primary outcome was the monthly dose of EPO and secondary outcome included dose of paricalcitol by the end of the study period and number of hospitalization. Records of the monthly quality assurance and performance improvement meetings were reviewed to accurately capture all hospitalizations. A visit to emergency room was not considered a hospitalization. Since the strongest association between 25(OH)D levels and outcomes has been seen in patients with severe VDD[8, 13], we performed a subgroup analysis in patients with 25(OH) D level <15 ng/ml. Based on the fact that vitamin D supplementation might improve erythropoiesis through better regulation of bone turnover with control of secondary hyperparathyroidism, a subgroup analysis was performed in patients in the D2 group who had the largest drop in PTH levels.

## Results

Table 2 shows the baseline characters of the study population including demographic, clinical history, laboratory parameters and monthly administered doses of iron sucrose, EPO, and paricalcitol. The patients in the control group were younger, had more coronary artery disease (CAD) and less cerebrovascular disease (CVD). The patients in the D_2_ group had higher 25(OH)D level, serum iron saturation (TSAT) and ferritin compared to the controls at baseline. Eighty-five percent of the study population consists of Hispanic race. As shown in Table 3, the D_2_ group experienced a significant increase in 25(OH)D levels from 16.2 ± 7.5 ng/ml at baseline to 30.6 ± 11.7 ng/ml at end of study, while levels remained low in the control group. Vitamin D sufficiency (25[OH]D > 30 ng/ml) was achieved in 45 % of the D_2_ group at 6 months and in 50 % at 12 months, while only 2.5 % of the control group was vitamin D sufficient at 12 months.

**Table 2:** Baseline Characteristics of The Study Population

	Total	Control	Ergocalciferol	*p* value
	(*n* = 186)	(*n* = 79)	(*n* = 107)	
Demographics (mean ± SD)				
Age, y	57.6 ± 13	55.2 ± 11.9	59.4 ± 13.2	0.01
Male, no. (%)	92 (49.5)	40 (50.6)	52 (48.6)	ns
Vintage, y	5.9 ± 4	6.3 ± 4.1	5.6 ± 3.2	ns
Co-Morbidities, number (%)				
Diabetes	128 (68.8)	51 (64.6)	77 (72)	ns
CAD	90 (48.4)	52 (65.8)	38 (35.3)	<0.001
Hypertension	172 (92.5)	72 (91.1)	100 (93.5)	ns
CVA	23 (12.4)	3 (3.8)	20 (18.7)	0.003
Cancer	7 (3.8)	1 (1.3)	6 (5.6)	ns
Laboratory Parameters (mean ± SD)				
Vitamin D level, ng/ml	16.8 ± 7	19.2 ± 6.8	16.2 ± 7.5	0.03
Hemoglobin, g/dl	11.1 ± 1	11.2 ± 1.1	11 ± 1.4	ns
TSAT, %	31.6 ± 16	27.5 ± 10	34.7 ± 18	<0.001
Ferritin, mg/dl	665 ± 383	538 ± 269	759 ± 425	<0.001
Calcium, mg/dl	9.1 ± 1	9.2 ± 0.7	9.1 ± 0.7	ns
Phosphorus, mg/dl	5.2 ± 2	5.1 ± 1.4	5.3 ± 1.7	ns
PTH, pg/ml median [IQR]	294 [178, 428]	283 [162, 408]	308 [184, 490]	ns
Albumin, mg/dl	4.1 ± 0.01	4.2 ± 0.4	4 ± 0.3	ns
Medications (median [IQR])				
Iron sucrose, mg/month	200 [0,287]	200 [25,400]	150 [0,250]	ns
EPO, IU/Month	26000 [12175, 48000]	26000 [12350, 49450]	27500 [13200, 48000]	ns
Paricalcitol, μg/month	26 [12, 39]	26 [10.5, 42]	25 [12.5, 39]	ns

**Table 3:** Primary and Secondary Outcomes and Related Variables at Different Time Points in 2 Groups

	Control Group				Ergocalciferol Group				*p* value Control vs ergo
Variable	Baseline	6 months	12 months	p for trend	Baseline	6 months	12 months	p for trend	
Vitamin D level, ng/ml	19.2 ± 6.8	19.7 ± 8.3	14.2 ± 9.3	0.002	16.2 ± 7.5	29.5 ± 9.9	30.6 ± 11.7	<0.001	<0.001
EPO dose, IU X 10^3^/month	26[12.3,49.4]	23.9[9.7, 42.2]	13[6.5,27]	0.01	27.5[13.2, 48]	26[16.4, 45.5]	23.4[9.5, 39]	0.02	ns
Hemoglobin, g/dl	11.2 ± 1.1	11.4 ± 1	10.7 ± 1.3	<0.001	11 ± 1.4	11.4 ± 1.5	10.9 ± 1.4	0.02	ns
TSAT, %	27.5 ± 10.1	31.6 ± 12.6	29.9 ± 10.9	0.03	34.7 ± 18	32.5 ± 12.2	31.7 ± 13.9	ns	0.02
Ferritin, mg/dl	538 ± 269	556 ± 260	614 ± 244	0.04	759 ± 425	691 ± 376	691 ± 370	ns	0.01
Albumin, mg/dl	4.2 ± 0.4	4.2 ± 0.2	4.2 ± 0.3	ns	4 ± 0.3	4.1 ± 0.3	4.3 ± 0.6	<0.001	<0.001
Iron sucrose, mg/month	260 ± 272	164 ± 151	199 ± 274	0.03	194 ± 231	180 ± 173	143 ± 162	ns	ns
Paricalcitol dose μg/month	26 [10.5, 42]	26[13, 51.5]	39[23.5, 52]	0.002	2512.5, 39]	24[12, 39]	20[9, 39]	ns	<0.001
Calcium, mg/dl	9.2 ± 0.7	9.2 ± 0.7	9.3 ± 0.8	ns	9.1 ± 0.7	9.4 ± 0.7	9.1 ± 0.8	<0.001	<0.001
Phosphorus, mg/dl	5.1 ± 1.4	5.6 ± 1.8	5.1 ± 1.3	0.01	5.3 ± 1.7	5.5 ± 1.7	5.7 ± 1.8	ns	0.03
PTH, pg/ml	283[162, 408]	253[151, 373]	257[160, 429]	ns	308[184, 490]	263[145, 416]	299[200, 471]	0.01	ns
Hospitalization n/year (%)	28 (35)				37 (35)				ns

Data is presented either as mean ± SD or median [IQR] depending on the distribution

### Erythropoietin dose requirement: primary outcome

**Fig. 1:**
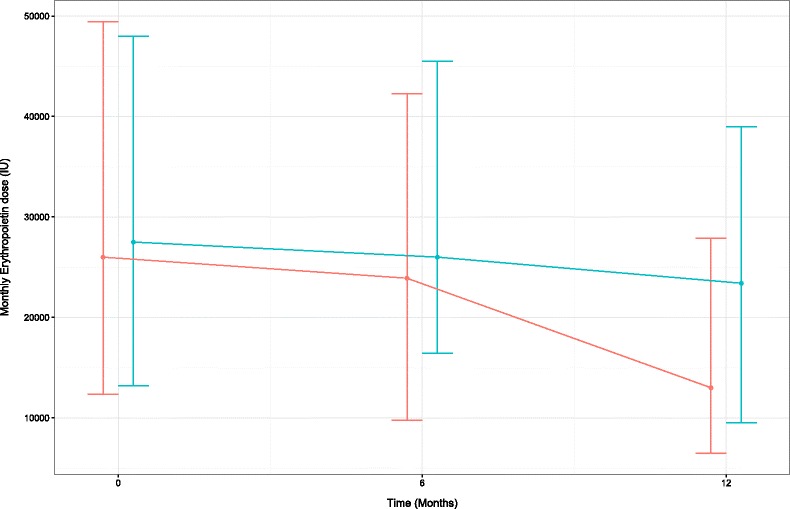
The monthly requirement of Erythropoietin dose (median [IQR]) over 1 year period in hemodialysis patients receiving ergocalciferol (*n* = 107, *blue*) or control (*n* = 79, *red*), *p* = ns

As shown in Table 3, there was no difference in administered EPO dose between the two groups at different time points (Fig. 1, univariate analysis). During the study, hemoglobin concentration decreased in both the groups without significant between-group difference; this drop in hemoglobin was driven by interim change of the CMS recommendations for hemoglobin target goal. Ferritin and TSAT increased in the controls and decreased in the D_2_ group over 12 months, and they tended to equalize between groups by study end, although in group comparisons both parameters remained significantly higher in the D2 group. Albumin levels increased in the intervention group over 1 year and despite starting from numerically lower baseline levels, they were higher than control at the end of the study. In univariate analysis, EPO dose correlated inversely with hemoglobin, ferritin and TSAT (all *p* < 0.001) and positively with iron sucrose treatment (*p* = 0.005), but it did not show association with ergocalciferol treatment or 25(OH)D levels, or other demographic and clinical variables. In multiple regression including all the above variables (with *p* < 0.1), the variation in EPO dose was explained by the levels of hemoglobin (p ≤ 0.001), ferritin (*p* = 0.004) and iron saturation (*p* = 0.01) but, notably, not by ergocalciferol treatment (Table 4).

**Table 4:** Multiple regression analysis within a linear mixed-effect model with EPO dose as dependent variable and other laboratory and clinical data entered

Variable	Estimate ± SE	*p* value
(Intercept)	110.2 ± 19.5	<0.001
Hemoglobin	−3.3 ± 0.9	<0.001
TSAT	−0.3 ± 0.1	0.004
Ferritin	−0.01 ± 0.0	0.01
Venofer treatment	0.01 ± 0.01	ns
Calcium	−2.7 ± 1.8	ns
Phosphorus	−0.7 ± 0.8	ns
CVA	−3.7 ± 6.6	ns
Effect of time at 6 months	0.13 ± 3.31	ns
Effect of time at 12 months	−7.50 ± 3.31	0.02
Effect of ergocalciferol at 6 months	0.96 ± 4.36	ns
Effect of ergocalciferol at 12 months	−0.84 ± 4.35	ns

### Paricalcitol dose requirement and hospitalization: secondary outcomes

**Fig. 2:**
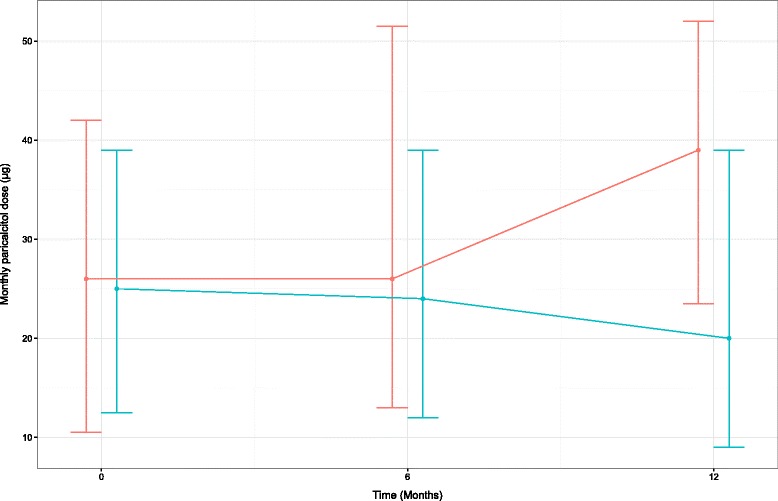
The monthly requirement of paricalcitol dose (median [IQR]) over 1 year period in hemodialysis patients receiving ergocalciferol (*n* = 107, *blue*) or control (*n* = 79, *red*), *p* < 0.001

The paricalcitol dose requirement increased substantially by the end of the study in the control group but remained stable in the D_2_ group (Fig. 2). The PTH levels were not different between groups even though overtime they decreased mildly but significantly in the D_2_ group (Table 3). By the end of the study, the D_2_ group had significantly higher serum phosphorus compared to control group. In univariate analysis, paricalcitol dose correlated directly with presence of diabetes (*p* = 0.02), CAD (*p* < 0.001) and with calcium levels (*p* = 0.02), but not with demographic variables, serum phosphorus, iPTH and 25(OH)D levels. In multiple regression that included all the above variables (with *p* < 0.1), the reduction in paricalcitol dose correlated with ergocalciferol treatment (*p* < 0.001; Table 5).

**Table 5:** Multiple regression analysis within a linear mixed-effect model with paricalcitol dose as dependent variable and other laboratory and clinical data entered

Variable	Estimate ± SE	*p* value
(Intercept)	0.5 ± 11.3	ns
Diabetes	6.6 ± 3.3	0.04
Cancer	14.3 ± 8.1	ns
CAD	10.1 ± 3.2	0.002
Calcium	2.2 ± 1.2	0.06
PTH	0.0 ± 0.0	ns
Effect of time at 6 months	2.8 ± 2	ns
Effect of time at 12 months	8 ± 2	<0.001
Effect of ergocalciferol at 6 months	−3.8 ± 2.8	ns
Effect of ergocalciferol at 12 months	−10.4 ± 2.8	<0.001

Approximately one third of all participants were hospitalized during the study period. The number of hospitalization correlated positively with the presence of diabetes and inversely with hemoglobin values; however, there was no difference in hospitalization rate in the ergocalciferol and control groups (Table 6).

**Table 6:** Multiple regression analysis within a linear mixed-effect model with hospitalization rate as dependent variable and other laboratory and clinical data entered

Variable	Log Odds ± SE	OR [CI95]	*p* value
Diabetes	1.2 ± 0.4	3.4 [1.5, 7.5]	0.003
Hypertension	1.0 ± 0.8	2.9 [0.6, 14.4]	ns
Hemoglobin	−0.4 ± 0.1	0.7 [0.5, 0.9]	0.009
25(OH)D level	0.006 ± 0.02	1.01 [0.9, 1.05]	ns
Ergocalciferol	−0.2 ± 0.3	0.8 [0.4, 1.5]	ns

### Follow-up cohort

**Fig. 3:**
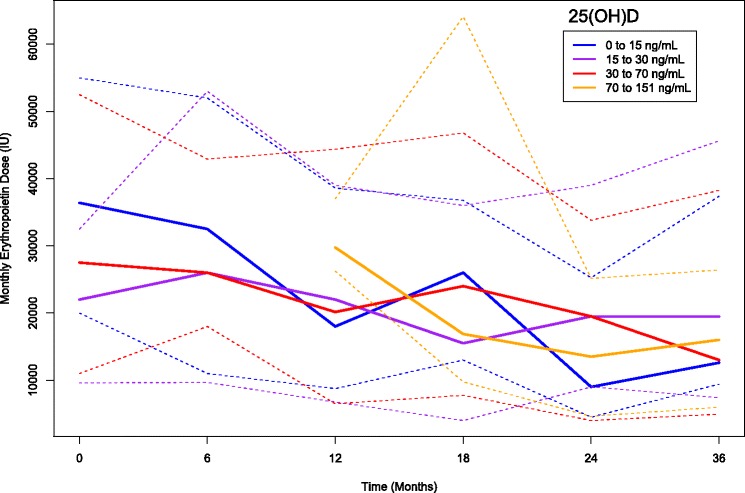
The univariate association of 25(OH)D levels (mean ± SD) with monthly EPO dose (median [IQR]) in the entire study population over 36 months of observation, *p* = ns

On univariate analysis, no relationship was found between the serum 25(OH)D levels and monthly EPO dose (Fig. 3).

### Subgroup analysis

#### 25(OH)D <15 ng/ml

Fifty patients in the D_2_ group and 40 in the control group started the study with 25(OH)D <15 ng/ml. Similar to the primary analysis, subanalysis of these subjects with severe VDD, showed no effect of ergocalciferol treatment on EPO requirements, but it associated with lower administration of paricalcitol during the period of observation. The hospitalization rate was the same between the subgroups.

#### D_2_ group with largest decrease in PTH levels

Twenty-seven of 107 patients in the D_2_ group had median drop of PTH (−221 pg/ml [IQR, −445, −120]), as compared to rest of the 80 patients (78 pg/ml [IQR, −14, 196]. The monthly EPO dose in these 27 patients did not change over 12 months of ergocalciferol supplementation (ΔEPO 230 IU/Month [IQR, −15500, 9200]), despite the fact that overall EPO requirement decreased in the study population due to CMS guideline changes.

## Discussion

In this study, administration of nutritional vitamin D2 did not result in reduction of EPO requirement in patients undergoing maintenance hemodialysis despite significant increase in 25(OH)D levels. Moreover, there was no reduction in the hospitalization rate; however, we observed a significant reduction in requirement of active vitamin D analogue dosage. In additional analyses, similar results were found in dialysis patients with severe VDD.

Our study is consistent with a recent randomized controlled trial of 6-month duration which showed no effect of nutritional vitamin D supplementation on EPO requirement [14]. We complement this information with an extended follow-up of 12 months in a study population consisting of 85 % Hispanics and 70 % diabetics. These results are of importance since VDD has been associated with many complications related to diabetes and there is lack of information on VDD in Hispanics [15, 16]. Our data, however, is at odds with previous investigations: Matias et al. reported 25 % reduction in use of darbepoetin after 6 months of cholecalciferol supplementation in 158 hemodialysis patients [2]; Rianthavorn et al. demonstrated reduction in ESA dose after 12 weeks of ergocalciferol administration in 10 CKD stage V patients aged <18 years [10]; in two other studies, 4–6 months of ergocalciferol administration reduced the dose of ESA in approximately 60 % of patients [11, 12]. Of note, all these studies had shorter duration, smaller sample size and lacked control group. We followed KDOQI guidelines to correct VDD in our dialysis population, where an oral weekly dose of 50,000 IU D2/D3 for 4–12 weeks is recommended for mild to severe vitamin D deficiency (Table 1). In our study, the recommended supplementation dose was sufficient to correct hypovitaminosis D in half of the patients on maintenance HD; however, the study leaves it unanswered whether higher dose or frequency of vitamin D may have resulted in higher rate of vitamin D sufficiency and decrease in EPO utilization in our patients. Nonetheless, we observed that the patients who achieved clearly high 25(OH)D levels also did not have lower EPO requirements (results not shown). Moreover, in a recently published study (DIVINE trial), 90 % of the HD patients achieved vitamin D sufficiency with 12 weeks of weekly 50,000 U of ergocalciferol treatment. Despite this rate of sufficiency, the authors did not report any difference in hemoglobin values or use of EPO in treatment and placebo arm over 12 weeks of treatment period [17].

Furthermore, consistent with the shorter duration DIVINE study [17], we did not find beneficial effects of vitamin D supplementation on the hospitalization rate in our patients. The effect of Vitamin D has not been previously reported on hospitalization in the chronic dialysis population in a longer study like ours [17], but the results of the present study are consistent with one conducted in critically ill non-ESRD patients, in whom administration of vitamin D did not reduce the lengths of ICU or hospital stay, the hospital mortality or the 6-month mortality [13]. Similar to previous studies [2, 18], we found that ergocalciferol administration reduced the need for active vitamin D analogues while the effects on serum calcium and phosphorus were negligible. We observed a significant decrease in iPTH in the treatment arm, but levels were not different compared to the control arm. These findings, however, remain difficult to be interpreted because we did not collect information about the use of phosphate binders and serum 1,25(OH)_2_D levels.

In many studies, the association between serum 25(OH)D levels and clinical outcomes was the strongest when the VDD was most severe. Based on this, we conducted additional analyses in the subgroup of patients with 25(OH)D level < 15 ng/ml, but found the same results as in the main study. Furthermore, over 2 years of additional observation, we did not find any relationship between vitamin D level and ESA dose, nor the duration of ergocalciferol treatment had any effect on ESA dosing.

Numerous mechanisms have been proposed to explain the potential benefit of vitamin D on erythropoiesis: a direct effect on erythroid precursor proliferation and/or an indirect one via reduction of inflammation and secondary hyperparathyroidism [19, 20]. In our study, we observed a significant increase in albumin in the treatment arm suggestive of reduction in inflammation; however, this did not associate with a decrease in ESA requirement. At our center, we prefer to reduce the dose of active vitamin D analogues rather than drastic decrease in iPTH levels to avoid vitamin D analogues associated complication such as hypercalcemia, hyperphosphatemia and adynamic bone disease while still maintaining iPTH levels within recommended range by KDIGO. This approach could have negated the beneficial effect of reduced iPTH level on ESA resistance. Although in a subanalysis of patients with the largest drop in iPTH level, we did not observe any reduction in ESA requirement. Nevertheless, our results are in alignment with recently published studies demonstrating no pleiotropic effects of nutritional D supplementation on the 24-h BP, arterial stiffness and cardiac function [21], or markers of cardiovascular risk, inflammation, muscle function or subjective health parameters in dialysis patients [22]; or chronic obstructive pulmonary disease (COPD) exacerbation and physical performance in COPD patients [23, 24], or asthma exacerbation [25].

Strengths of our study include the direct administration of intervention by the clinic staff ensuring 100 % adherence, presence of a control arm, long duration of follow-up, relatively large sample size and the very conspicuous differences in 25(OH)D status between groups. The major flaw of the study was the change in the target hemoglobin recommended by CMS in the middle of the study, even though the rate of drop in the hemoglobin was not different in the two groups. Due to retrospective nature of the study there were many significant confounding factors. The multivariable analysis could ameliorate these factors to some extent; however, is not infallible. Additionally, we could not account for residual confounding. Lastly, the majority of patients were Hispanic; therefore, our study population was not necessarily representative of the general North-American HD population. Nevertheless, our study adds invaluable information to the emerging literature failing to demonstrate the beneficial effect of vitamin D supplementation in different populations.

## Conclusion

The supplementation of vitamin D deficiency with ergocalciferol is a simple and cost effective measure but we were unable to demonstrate its benefit on EPO requirement and hospitalization rate in hemodialysis patients. There is a need for large prospective trials to support the KDIGO guidelines to routinely supplement this population with nutritional vitamin D.

## Abbreviations

1,25(OH)2D: 1,25-dihydroxy vitamin D
25(OH)D: 25-hydroxyvitamin D
CAD: Coronary artery disease
CKD: Chronic kidney disease
CMS: Centers of Medicare and Medicaid services
COPD: Chronic obstructive pulmonary disease
CVD: Cerebrovascular disease
D2: Ergocalciferol
EPO: Erythropoietin
ESA: Erythropoietin simulating agents
ESRD: End-stage renal disease
HD: Hemodialysis
iPTH: intact parathyroid hormone
IQR: Interquartile ranges
KDIGO: Kidney Disease Improving Global Outcome
SD: Standard deviation
TSAT: Iron saturation
VDD: Vitamin D Deficiency
